# Expression of activated signal transducer and activator of transcription-3 as a predictive and prognostic marker in advanced esophageal squamous cell carcinoma

**DOI:** 10.1186/s12957-015-0726-z

**Published:** 2015-11-09

**Authors:** Chuying Huang, Li Wang, Xibiao Yang, Lin Lai, Dian Chen, Chunyan Duan

**Affiliations:** Department of Respiration, Affiliated Hospital of Hubei Institute for Nationalities, Enshi, Hubei Province China; Department of Medical Oncology, Enshi Tujia and Miao Autonomous Prefecture Central Hospital, No. 158, Wu Yang Avenue, Enshi, Hubei Province China; Department of Dermatology, Enshi Tujia and Miao Autonomous Prefecture Central Hospital, No. 158, Wu Yang Avenue, Enshi, Hubei Province China; Department of Radiology, West China Hospital of Sichuan University, No. 37, Guo Xue Xiang, Chengdu, Sichuan China

**Keywords:** Esophageal squamous cell carcinoma, p-Stat3 expression, Chemotherapy

## Abstract

**Background:**

Signal transducer and activator of transcription-3 (STAT3) is an oncogenic transcription factor constitutively active and aberrantly expressed in various types of malignancies, and the expression of p-STAT3 has been recognized as a predictor of poor survival. It remains unclear how variations in p-STAT3 expression influence clinical outcomes in esophageal squamous cell carcinoma (ESCC).

**Methods:**

Between 1 January 2008 and 1 November 2013, 153 advanced esophageal squamous cell carcinoma patients (stage IV) from two cancer centers in West China were treated with paclitaxel and cisplatin. We retrospectively analyzed the clinical outcomes of patients with ESCC and examined the correlation between p-STAT3 levels and clinical outcomes in esophageal cancer patients.

**Results:**

Among the 153 patients, positive p-STAT3 expression was observed in 73 of 153 (47.7 %) cases. The median PFS for patients with positive expression of p-STAT3 and negative expression of p-STAT3 was 5.0 months and 6.9 months, respectively (*P* < 0.001). The median overall survival was significantly higher in patients with p-STAT3 negative tumors than in those with p-STAT3 positive tumors (9.9 vs 8.9 months, *P* = 0.026). Kaplan–Meier survival analysis showed that p-STAT3 expression was statistically indicative of a poor prognosis for progression-free survival.

**Conclusions:**

These data showed that p-STAT3 expression was significantly associated with poor prognosis in patients with esophageal cancer and could be used as a predictive and prognostic marker in esophageal cancer.

## Background

The incidence of esophageal adenocarcinomas predominantly affecting the lower esophagus and gastroesophageal junction has increased substantially in USA and the Europe, whereas the majority of esophageal cancers worldwide are of the squamous cell carcinoma type, the most common histological type in China [1]. Esophageal cancer is an aggressive upper gastrointestinal malignancy which generally presents as a locally advanced tumor that requires multimodal therapy, and the prognosis in patients with esophageal cancer remains poor, with a 5-year survival of 15–34 % [2–4]. Identification of the potential molecular markers for predicting the treatment response and prognosis of esophageal cancer is very important. Currently, much interest has been focused on the signal transducer and activator of transcription-3 (STAT3), a member of the Janus-activated kinase/STAT signaling pathway.

STAT3 can be activated by diverse upstream kinases including cytokine receptors and tyrosine kinases, and STAT3 was activated in nearly 50 % of the squamous cell carcinoma [5]. Constitutive activation of STAT3 has been shown in various types of malignancies, and the expression of p-STAT3 has been recognized as a predictor of poor survival [5–8]. In esophageal cancer, however, little is known about the prognostic relevance of p-STAT3, although Chen et al. [5] showed the expression of p-STAT3 in an immunohistochemical study. We therefore investigated the correlation between p-STAT3 levels and clinical outcomes in esophageal cancer patients.

## Methods

One hundred and fifty-three advanced esophageal squamous cell carcinoma patients (stage IV) were treated with paclitaxel and cisplatin between 1st January 2008 and 1st October 2013, at West China Hospital, Sichuan University, and the Central Hospital of Enshi Tujia and Miao Autonomous Prefecture. The main end points were progression-free survival (PFS), overall survival (OS), and response to chemotherapy. PFS was defined as the lapse of time between the start of chemotherapy and progressive disease or death. OS was defined as the lapse of time between the start of chemotherapy and death of any cause. The study protocol was approved by the Institutional Review Board of Enshi Tujia and Miao Autonomous Prefecture Central Hospital.

During treatment, all patients underwent chest CT scan every 2–3 months as the part of clinical routine practice. PFS and OS distributions were estimated using the Kaplan–Meier method, and survival curves were compared by log-rank test. Univariate and multivariate Cox proportional hazards regression analysis was performed to determine independent prognostic factor for disease survival. All the parameters related to PFS and OS (by Kaplan–Meier with a *P* value <0.2) were included into the multivariate model (Cox regression). A *p* value of less than 0.05 was considered statistically significant. Data was analyzed by using the SPSS 16.0 (SPSS Inc., Chicago, Illinois) software package. Adverse events were classified according to the National Cancer Institute Common Terminology Criteria for Adverse Events (NCI-CTCAE) version 4.0.

### Immunohistochemical staining (IHC)

Sections (3 mm thick) of formalin-fixed, paraffin-embedded tumor specimens were heated in a microwave oven to maintain the temperature at 92–98 °C for 10–15 min for antigen retrieval, then incubated with 10 % normal goat serum. The sections were first incubated with primary purified rabbit antihuman STAT3 polyclonal antibody (1:150, Santa Cruz Biotech, CA, USA) at 4 °C overnight, then biotin-labeled rabbit anti-goat secondary antibody at room temperature for 30 min. After washing with PBS three times, streptavidin-coupled horseradish peroxidase (streptavidin-HRP) was added and incubated at room temperature for 20 min, followed by adding freshly prepared 0.01 % diaminobenzidine with 5–10 min incubation. The reaction was terminated by washing with distilled water three times. Cells were counterstained with hematoxylin–eosin and returned to blue with ammonia water. After staining, the sections were dehydrated with gradient ethanol, cleared with xylene, and then mounted with the cover slips.

STAT3 expression level was determined by integrating the percentage of stained cells and staining intensity (bordering (score 1), weak (score 2), moderate (score 3), and strong (score 4)). The extent of staining was scored according to the percentage of positive-stained tumor cells in the field: Score 0 was attributed to tumors with absence of staining. The extent of staining was scored as 0 (negative), 1 (0–25 %), 2 (26–50 %), 3 (51–75 %), or 4 (76–100 %), according to the percentage of positive-stained tumor cells in the field. The product of the intensity and extent score was considered as the mean IHC score. The staining was observed and assessed by three independent pathologists blind to clinical data.

## Results and discussion

### Expression of p-STAT3 in esophageal squamous cell carcinoma tissue

**Fig. 1:**
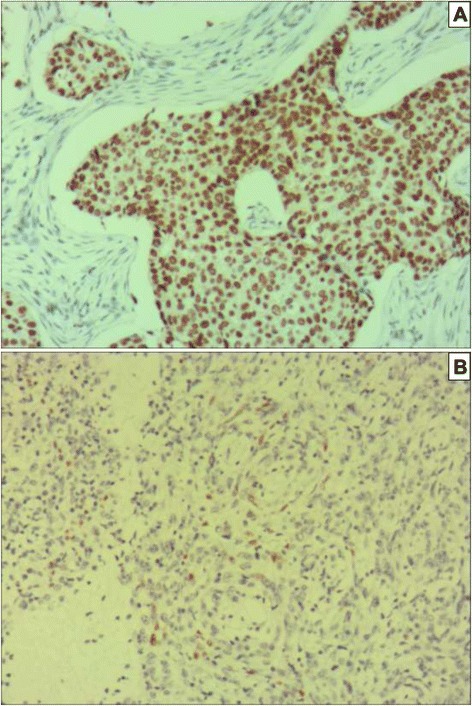
IHC pictures: **a. **Predominantly the cytoplasmic staining of p-STAT3 in esophageal squamous cell cancer (SP method, ×100). **b.** p-STAT3 negative expression in esophageal squamous cell cancer (SP method, ×100)

Phosphorylated-STAT3 was expressed only in the nuclei of the tumor cells [9] (Fig. 1a, b), and our results showed that elevated levels of p-STAT3 protein were detected in the nuclei of 73 out of 153 total esophageal squamous cell carcinoma cancer specimens.

### Prognostic significance of p-STAT3 expression

**Fig. 2:**
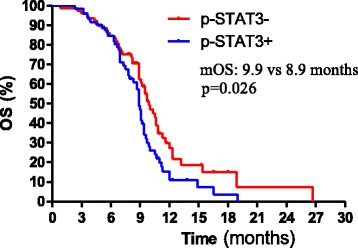
Kaplan–Meier curves showing median progress free survival (mPFS) for 153 metastasis esophageal cancer patients treated with paclitaxel and cisplatin according to p-STAT3 levels. The median PFS for patients with positive expression of p-STAT3 and negative expression of p-STAT3 was 5.0 months and 6.9 months, respectively (HR 1.380, 95 % CI 0.698 to 2.062, *p* < 0.001)

**Fig. 3:**
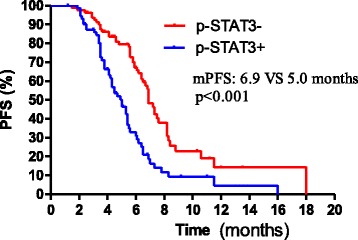
Kaplan–Meier curves showing median overall survival (mOS) for 153 metastasis esophageal cancer patients treated with paclitaxel and cisplatin according to p-STAT3 levels. The median overall survival was significantly higher in patients with p-STAT3 negative tumors than in those with p-STAT3-positive tumors (9.9 vs 8.9 months, HR 1.112, 95 % CI 0.4305 to 1.794, *p* = 0.026)

One hundred and fifty-three advanced esophageal squamous cell carcinoma patients started paclitaxel (135 mg/m^2^) and cisplatin (75 mg/m^2^) as the first line treatment between 1st January 2008 and 1st November 2013. Median follow-up time was 8.6 months (8.6 ± 3.5, range 2.6–26.7). Among the 153 patients, 80 (52.3 %) were negative, and 73 (47.7 %) were positive for p-STAT3 expression. There were no significant correlations between p-STAT3 expression levels and clinical characteristics, including age (*P* = 0.605), gender (*P* = 0.816), differentiation (*P* = 0.599), site of metastasis (*P* = 0.298), or site of tumor (*P* = 0.415, Table 1). The median PFS for patients with positive expression of p-STAT3 and negative expression of p-STAT3 was 5.0 months and 6.9 months, respectively (HR 1.380, 95 % CI 0.698 to 2.062, *p* < 0.001, Fig. 2). The median overall survival was significantly higher in patients with p-STAT3-negative tumors than in those with p-STAT3-positive tumors (9.9 vs 8.9 months, HR 1.112, 95 % CI 0.4305 to 1.794, *p* = 0.026, Fig. 3). Table 2 gives an overview of all previously described prognostic factors assessed by univariate analysis. In univariate, primary site of tumor, p-STAT3 expression levels, and chemotherapy cycles were significant in univariate analysis. In multivariate analysis (Table 3), primary site and p-STAT3 expression levels were independently linked to PFS. Degree of differentiation and primary site were an independent prognostic factor in multivariate analysis for disease overall survival.

**Table 1:** Patient characteristics

		p-STAT3+	p-STAT3−	*P*
Age (year)				
Mean	40-83	40-83	42–81	0.605
Range	58.100	58.100		
Sex				
Male	127.000	61.000	66	0.816
Female	26.000	12.000	14	
Primary sit				
Upper thorax	26.000	12.000	14	
Middle thorax	46.000	22.000	24	0.415
Lower thorax	81.000	39.000	42	
Degree of differentiation				
Well	35.000	16.000	19	
Moderate	74.000	34.000	40	0.599
Poor	44.000	23.000	21	
Local/distant metastasis				
Local	17.000	5.000	11	0.298
Distant	137.000	68.000	69	
Site of metastasis				
Lung	49.000	20.000	29	
Liver	25.000	15.000	10	
Lymph node	68.000	32.000	36	0.307
Lung and liver	6.000	4.000	2	
Other	5.000	2.000	3

**Table 2:** Univariate analysis

Patient characteristics	PFS	p	OS	p
Age (years)				
*v* < 60	6.2	0.08	9.5	0.929
≥ 60	5.8		9.1	
Sex				
Female	5.3	0.584	9	0.949
Male	6.2		9.4	
Degree of differentiation				
Well	6.8	0.08	10	0.119
Moderate	6.4		9.2	
poor	5.5		8.9	
Primary site				
Upper thorax	7.3	0.001	9.2	0.038
Middle thorax	6.7		10.3	
Lower thorax	3.8		8.9	
Chemotherapy cycles				
1	6.1	0.145	8	0.038
2	5.6		8.2	
3	6.4		9.2	
≥ 4	6.7		10.5	
Metastasis				
Local	6.9	0.0587	9.5	0.714
Distant	5.9		9.2

**Table 3:** multivariate analysis

For PFS	*P* value	Hazard ratio	95 % CI
Age	0.947	1.014	0.674–1.524
Chemotherapy cycles	0.531	1.063	0.879–1.285
Primary site	0.001	1.839	1.296–2.610
Degree of differentiation	0.809	1.031	0.808–1.315
Local/distant metastasis	0.478	1.238	0.687–2.229
p-STAT3 expression levels	0.009	0.554	0.356–0.862
For OS	*P* value	Hazard ratio	95 % CI
Age	0.810	0.899	0.598–1.352
Chemotherapy cycles	0.899	1.012	0.844–1.212
Primary site	0.050	1.431	0.999–2.049
Degree of differentiation	0.041	0.761	0.586–0.990
Local/distant metastasis	0.302	1.380	0.748–2.543
p-STAT3 expression levels	0.161	0.732	0.473–1.132

### Response to chemotherapy in esophageal squamous cell carcinoma

Response results are summarized in Table 4. Of the 73 patients with positive expression of p-STAT3, 9 (12.3 %) achieved a complete response (CR), 32 (43.8 %) achieved a partial response (PR). Seventy patients demonstrated a stable disease (SD), which resulted in a response rate of 56.1 %. For those patients with negative p-STAT3 expression, 12 achieved a confirmed response, 36 were partial responses, and 18 had stable disease. There were no significant differences in RR rate between patients with positive p-STAT3 expression and negative p-STAT3 expression (*p* = 0.264). Table 5 lists the adverse events and the proportion of patients experiencing adverse events during the treatment. The common grade 3⁄4 adverse events were anemia, neutropenia, thrombocytopenia, vomiting, constipation, anorexia, nausea, and leukocytopenia.

**Table 4:** Reponses to chemotherapy in the two group cases (%)

GROUP	RR	CR	PR	SD	PD
p-STAT3+	41 (56.1 %)	9 (12.3 %)	32 (43.8 %)	17 (22.5)	15 (20.6 %)
p-STAT3−	52 (65.0 %)	12 (15 %)	36 (45.0 %)	18 (23.8)	14 (17.5 %)
*P* value	0.264	0.632	0.491	0.885	0.631

**Table 5:** Events in the two groups (cases %)

Item	All grades		Grade 3⁄ 4	
	p-STAT3+	p-STAT3−	p-STAT3+	p-STAT3−
Leukocytopenia	49 (67.1 %)	60 (75 %)	7 (9.6 %)	8 (10 %)
Neutropenia	60 (82.2 %)	63 (78.7)	13 (17.8 %)	15 (18.8 %)
Hemoglobin	44 (60.3 %)	49 (61.2 %)	22 (30.1 %)	20 (25 %)
Thrombocytopenia	13 (16.3 %)	14 (17.5 %)	10 (13.7 %)	10 (12.5 %)
Nausea	7 (9.6 %)	9 (11.2 %)	8 (11 %)	9 (11.25 %)
Vomiting	13 (16.3 %)	15 (18.6 %)	12 (16.4 %)	13 (16.25 %)
Anorexia	14 (19.2)	11 (13.8 %)	9 (12.3 %)	9 (11.25 %)
Diarrhea	1 (1.4 %)	1 (1.3 %)	0	0
Constipation	16 (21.9 %)	19 (23.8 %)	10 (13.7 %)	15 (18.8)
Fatigue	31 (42.5 %)	30 (37.5)	3 (4.1 %)	5 (6.3 %)
Liver dysfunction	14 (19.1 %)	17 (21.3 %)	4 (5.5 %)	6 (7.5 %)

### Discussion

The prognosis for patients with advanced esophageal carcinoma is poor because of the limited response to conventional chemotherapeutic agents. The combination of docetaxel with cisplatin was reported in a phase II study has a response rate of 43 % in patients with gastric or gastroesophageal cancer. The median overall survival time on this study of 8.9 months in patients with positive expression of pSTAT3 and 9.9 months in p-STAT3 negative group is comparable to the results in phase II and III studies testing platinoid-based combinations [10–12]. Nevertheless, by far, there are no efficient molecular markers to predicting the treatment response and prognosis of esophageal cancer.

Immunoexpression of p-STAT3 has been recognized as a predictor of poor survival in many malignancies [6, 7, 13]. Immunohistochemical data [9] showed that p-STAT3 was significantly overexpressed in esophageal squamous cell carcinoma (ESCC) when compared with those in normal esophageal mucus, and overexpression of STAT3 significantly associated with the metastasis of ESCC through increasing the invasive ability of tumor cells. In this study, we found that the expression of activated STAT3 (p-STAT3) significantly correlated with a worse prognosis in patients with this disease by univariate analysis. p-STAT3 expression was an independent prognostic factor for progression-free survival; it was indicated that p-STAT3 expression can be an important factor to predict prognosis. One immunohistochemical study [14] has been reported on p-STAT3 expression in esophageal carcinoma, in which the researchers reported that 82 specimens (47.4 %) showed positive immunoexpression of p-STAT3 in 173 esophageal cancer specimens. Similarly, STAT3 was activated in 47.7 % of esophageal squamous cell carcinoma in immunohistochemical analysis of our study. In univariate and multivariate analyses, poor treatment response, no tumor resection, and positive IL-6 staining were significantly associated with shorter survival. However, there were no details about whether positive STAT3 staining was associated with shorter survival in the study. Another study by Li and co-workers [15] demonstrated that high expression of STAT3 was detected in 54 out of 82 TMA cancer tissues (65.9 %) while most of the adjacent non-cancerous tissues showed low expression of STAT3. More importantly, high expression of STAT3 was found to have a highly significant relationship with advanced tumor stage (*P* = 0.047) and poor prognosis (*P* = 0.023). The median overall survival time was 27.67 months in patients with low expression of STAT3 compared with 17.48 months in patients with high expression of STAT3 (*P* = 0.044). Similarly, in our study, we determined that high expression of STAT3 significantly correlated with poor prognosis. The median overall survival was significantly higher in patients with pSTAT3-negative tumors than in those with p-STAT3-positive tumors (9.9 vs 8.9 months, HR 1.112, 95 % CI 0.4305 to 1.794, *p* = 0.026, Fig. 3). However, we found the median overall time in our study was obviously shorter than that in Li’s study. We thought the cause might be the tumor stages, of 82 patients, 28 (34.1 %) had a stage I + II diagnosed at surgery in Li’s study.

STAT proteins are a family of latent cytoplasmic transcription factors and contribute to the progression of malignant transformation [16]. The STAT family of transcription factors is involved in several cellular processes, such as proliferation, survival, apoptosis, and differentiation [17, 18]. STAT3 protein was overexpressed and persistently activated appears to play a role in the cell transformation and tumor progression by stimulating cell growth, promoting tumor angiogenesis, mediating immune evasion, and conferring resistance to apoptosis induced by chemotherapeutic agents [19–21]. Although our result indicated that the activation of STAT3 contributed to a poor prognosis, the mechanism of constitutive activation of STAT3 has not been clarified well in esophageal squamous cell carcinoma. It is reported that STAT3 activated by diverse receptor and non-receptor tyrosine kinases, including, platelet-derived growth factor receptor, EGFR, Janus-activated kinases (JAK), and src in other malignancies [22]. STAT3 is an important mediator of the oncogenic effects of EGF and transforming growth factor in squamous cell carcinoma of the head and neck and non-small lung cancer [22]. Chen et al. [5] showed that apoptosis had been induced by the inhibition of STAT3 with a small molecular inhibitor of the Janus-activated kinase/STAT pathway in cervical cancer. Chen W et al. [23] demonstrated that addition of a STAT3 inhibitor, NSC-74859, to a standard regimen of cetuximab can increase the susceptibility of hepatoma cell lines to cetuximab. It is reported that combined inhibition of EGFR and STAT3 by erlotinib and niclosamide more effectively induced apoptosis in tumor tissues without toxicity for normal tissues [24]. STAT3 signaling pathways play a pivotal role in oncogenesis, and thus targeting the STAT signaling pathway appears to be an effective anticancer treatment strategy.

In summary, our results demonstrated that p-STAT3 expression in esophageal cancer acts as a predictor of poor prognosis. STAT3 appears to be one of the oncogenic pathways activated in human esophageal cancers. The constitutive STAT3 signaling may be a novel therapeutic target for esophageal cancers.

## Conclusions

These data showed that p-STAT3 expression was significantly associated with poor prognosis in patients with esophageal cancer, and could be used as a predictive and prognostic marker in esophageal cancer.

## Abbreviations

ESCC: esophageal squamous cell carcinoma
STAT3: signal transducer and activator of transcription-3
CR: complete response
PR: partial response
SD: stable disease
